# A neural stem cell–derived 3D spheroid model that recapitulates prion infection and pathology

**DOI:** 10.1016/j.mtbio.2026.103378

**Published:** 2026-06-22

**Authors:** Hyun Jung Park, Hyeri Kim, Sanghoon Byun, Chongsuk Ryou

**Affiliations:** a Department of Pharmacy, College of Pharmacy, and Institute of Pharmaceutical Science & Technology, Hanyang University ERICA, 55 Hanyangdaehak-ro, Ansan, Gyeonggi-do, 15588, Republic of Korea; b Department of Health Science and Technology, Samsung Advanced Institute of Health Sciences and Technology (SAIHST), Sungkyunkwan University, Republic of Korea

**Keywords:** Prion disease, 3D modeling, Neural stem cells, Novel platform, Therapeutic screening

## Abstract

Prion diseases are fatal, transmissible neurodegenerative disorders marked by the accumulation of misfolded prion protein (PrP^Sc^) in the central nervous system. Most in vitro models, largely 2D cultures, have been used to study prion biology but often fail to capture the brain's structural and cellular complexity. Here, we present a 3D spheroid model of neurons and astrocytes derived from mouse neural stem cells (NSCs) to study prion pathogenesis. NSCs were induced to form spheroids and then infected with Rocky Mountain Laboratory prions. Infected spheroids exhibited apoptosis and gliosis, recapitulating aspects of a neuron–astrocyte microenvironment and enabling the observation of neurodegenerative processes. We also show that the spheroids propagate prions, retain infectivity, and preserve pathological and cellular features after cryopreservation. This model supports sustained observation of prion dynamics over the tested culture period and assessment of therapeutic agents, bridging the gap between simple in vitro systems and in vivo models. Our findings highlight this 3D platform as a useful tool for prion research.

## Highlights

• A 3D neuron–astrocyte spheroid model supports robust and reproducible prion infection.
• Prion-infected spheroids exhibit apoptosis, neuronal loss, and pronounced astrogliosis.
• RT-QuIC shows strong and consistent PrP^Sc^ seeding activity across spheroid replicates.
• Spheroid-derived prions retain infectivity and transmit to naïve N2a cells.
• Cryopreserved infected spheroids maintain their structure and prion seeding capacity.

## Graphical abstract

**Figure:**
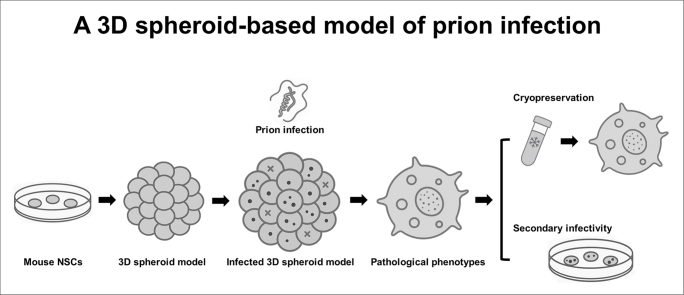

## 1 Introduction

Prion diseases are a group of fatal, progressive neurodegenerative disorders that primarily affect the central nervous system (CNS) [1]. They include Creutzfeldt–Jakob disease and kuru in humans, scrapie in sheep and goats, and bovine spongiform encephalopathy in cattle [2]. Prion diseases are caused by proteinaceous agents, termed prions [3]. These unconventional infectious pathogens are composed of the scrapie prion protein (PrP^Sc^), the misfolded isoform of the normal cellular prion protein (PrP^C^) [4]. PrP^Sc^ is rich in β-sheet structure and prone to aggregation [5]. The hallmarks of prion pathology include neuronal loss, astrogliosis, spongiform degeneration, and PrP^Sc^ accumulation within the brain [6].

Numerous animal and cellular models have been developed to understand prion transmission and pathogenesis [7]. Animal models, particularly rodent models, provide valuable insights into prion infectivity and disease progression in vivo [8]. However, they are costly, time-consuming, and often unsuitable for high-throughput mechanistic studies due to ethical constraints and long incubation periods [9,10]. In contrast, cell-based models, especially those using neural stem cells (NSCs), offer the advantages of reproducibility, scalability, and excellent control over experimental conditions. NSCs possess self-renewal capacity and can differentiate into neurons, astrocytes, and oligodendrocytes, making them a suitable platform for studying prion-induced cytotoxicity [11]. Previous studies have shown that both undifferentiated and differentiated NSCs, also called neurospheres, are susceptible to prion infection and can replicate disease-associated phenotypes such as PrP^Sc^ accumulation and neurotoxicity [[12], [13], [14]].

However, the NSC-based models are generally constrained by a lack of organized tissue structure, the absence of multicellular interactions, and limited ability to sustain prion replication over extended periods. To overcome those limitations, interest in establishing standardized and scalable 3D stem cell–based culture systems has been increasing to more faithfully mimic the cytoarchitecture and cellular diversity of the CNS and enable pharmacological screening [15]. Such models are essential for improving the reproducibility of experimental results and enhancing understanding of prion propagation in a controlled setting.

In this study, we developed a three-dimensional (3D) spheroid co-culture system in a 96-well plate format with defined differentiation conditions for NSC-derived neurons and astrocytes. This model supports stable neural lineage differentiation and is permissive to prion infection. Compared with previous 2D culture systems and animal models, which are limited by physiological relevance, scalability, and ethical constraints, our 3D spheroid platform provides a more physiologically relevant neuron–astrocyte microenvironment for studying prion-associated cellular interactions. By incorporating a simple and robust real-time quaking-induced conversion (RT-QuIC)-based detection method to monitor PrP^Sc^ accumulation and a standard scrapie cell assay to quantify prion infectivity in small spheroids, we established a practical and reproducible platform for prion research. This system not only enables the investigation of prion pathogenesis but also offers a scalable, high-throughput method for screening potential anti-prion agents, thereby addressing key limitations of existing models.

## 2 Results

### 2.1 Prion infection of murine NSC–derived cells in a 2D cell culture system

**Fig. 1:**
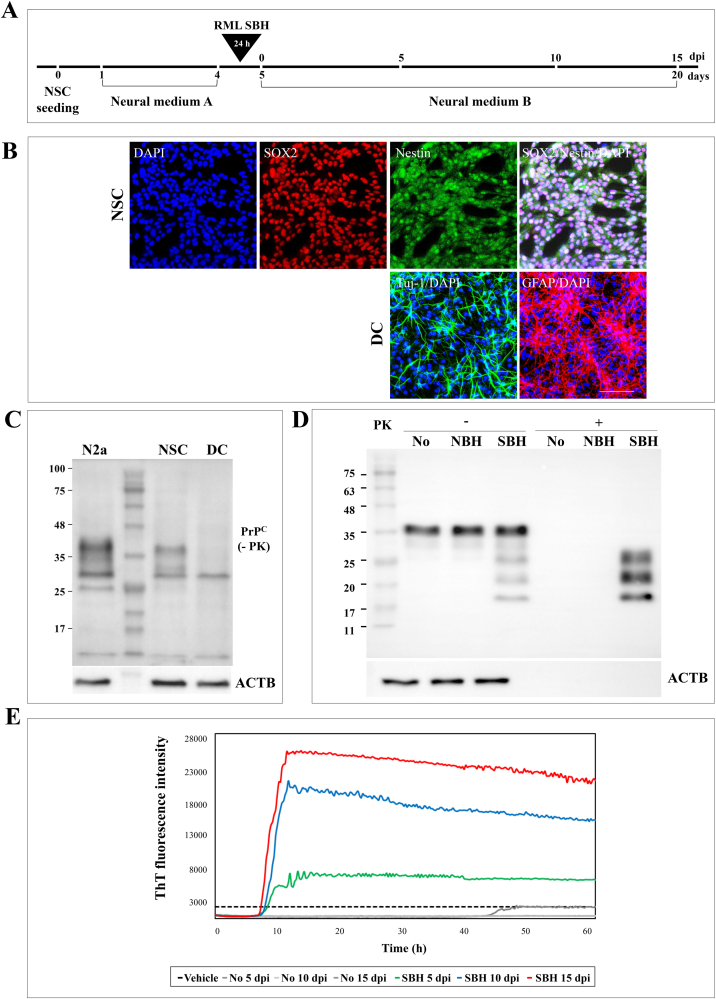
**Characterization of neural stem cells, differentiated cells, and prion infection in the NSC-derived 2D culture system.** (**A)** Schematic overview of the experimental timeline for neural differentiation and prion infection. NSCs were seeded and pre-cultured in neural medium A, followed by exposure to RML scrapie brain homogenate (SBH) for 24 h. After inoculation, cells were maintained in neural medium B and collected at the indicated time points. **(B)** Immunofluorescence staining of undifferentiated NSCs and differentiated cells (DCs). Undifferentiated NSCs expressed the neural stem cell markers SOX2 and Nestin, whereas differentiated cells expressed the neuronal marker Tuj-1 and the astrocytic marker GFAP. Nuclei were counterstained with DAPI. **(C)** Western blot analysis of cellular prion protein (PrP^C^) expression in N2a cells, NSCs, and differentiated cells without proteinase K (PK) treatment. ACTB was used as a loading control. (D**)** Western blot analysis of samples with and without PK treatment. In the absence of PK, PrP signals were detected in the no-treatment (No), normal brain homogenate-treated (NBH), and scrapie brain homogenate-treated (SBH) groups. After PK treatment, PK-resistant PrP^Sc^ was detected only in the SBH-treated group. ACTB was used as a loading control. **(E)** RT-QuIC analysis of lysates collected from 2D cultures at 5, 10, and 15 days post-inoculation (dpi). Thioflavin T (ThT) fluorescence increased over time in the infected groups, indicating progressive prion seeding activity. Vehicle-treated samples served as a negative control, and SBH samples served as positive controls. All scale bars: 100 μm.

Before we established a prion-infected 3D spheroid model, we examined whether differentiated cells derived from the NSCs of wild type (FVB) mice could be infected with prions in a 2D cell culture system. Prior to prion infection, we examined the characteristics of the NSCs and their differentiated neurons and astrocytes. The NSCs were differentiated into neurons and astrocytes over 20 days in neural differentiation media that included nerve growth factor (NGF) (Fig. 1A). Immunofluorescence microscopy showed that undifferentiated NSCs expressed the NSC markers Sox2 and Nestin, and after neural differentiation, they expressed a neuronal marker (Tuj-1) and an astrocyte marker (glial fibrillary acidic protein (GFAP)) (Fig. 1B). Because prion replication requires the expression of host PrP^C^ [16], we investigated the PrP^C^ expression of the NSCs and their differentiated cells. Western blotting showed the typical PrP^C^ band pattern with various glycosylation states in the NSCs and a single band PrP^C^ in its predominantly unglycosylated state in the differentiated cells (Fig. 1C). Next, we infected the NSCs with prions (Fig. 1A). After NSC seeding, the cells were cultured for 3 days in neural medium A and then exposed to 10% brain homogenate from Rocky Mountain Laboratory (RML)-infected mice in neural medium B for 24 h. The medium was then replaced with fresh neural medium B, and the cells were cultured for an additional 15 days with daily medium changes. Western blot analysis of cells harvested 15 days post-inoculation (dpi) demonstrated an accumulation of proteinase K (PK)-resistant PrP^Sc^ (Fig. 1D). Additionally, in RT-QuIC analyses of seeds obtained 5, 10, and 15 dpi, thioflavin T (ThT) fluorescence intensity increased over time, suggesting that robust PrP^Sc^ propagation occurred during NSC differentiation into neurons and astrocytes (Fig. 1E).

### 2.2 Dimensional shrinkage, cytotoxicity, gliosis, and neuronal degeneration in NSC-derived 3D spheroids infected with prions

**Fig. 2:**
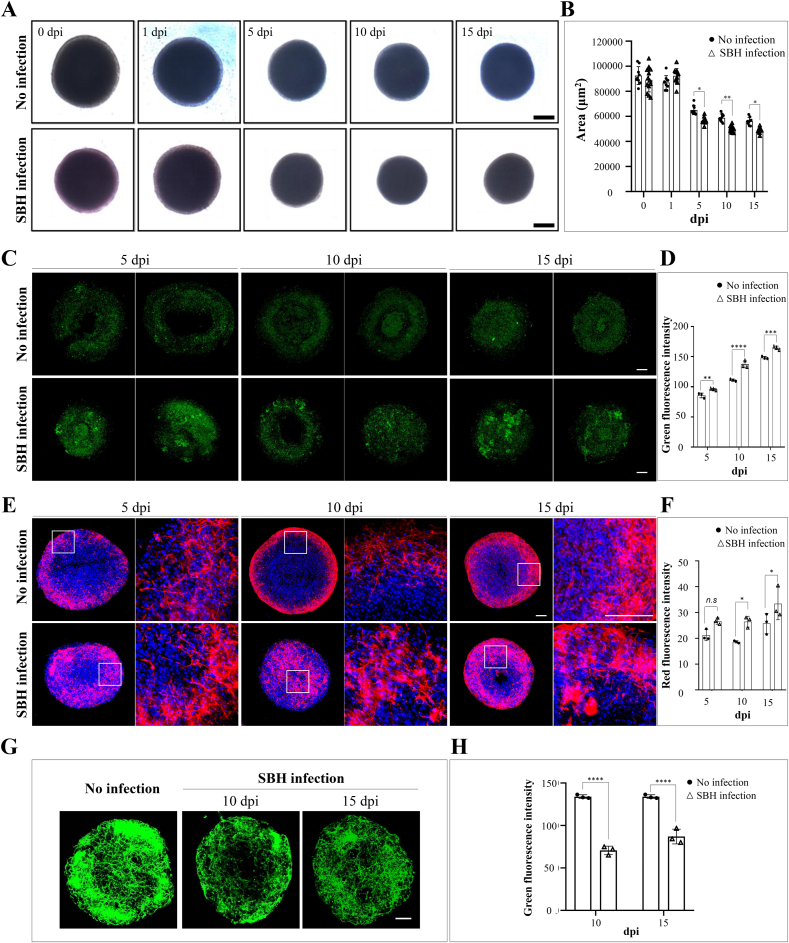
**Dimensional shrinkage, apoptosis, astrogliosis, and neuronal degeneration in prion-infected NSC-derived 3D spheroids.** (A) Representative bright-field images of non-infected and scrapie brain homogenate (SBH)-infected 3D spheroids collected at 0, 1, 5, 10, and 15 days post-inoculation (dpi). SBH-infected spheroids exhibited marked shrinkage over time compared with non-infected controls. **(B)** Quantification of spheroid area at the indicated time points. SBH-infected spheroids showed a significant reduction in area relative to non-infected spheroids, with the most pronounced decrease observed during the early post-inoculation period. **(C)** Representative fluorescence images of caspase-3/7 activity in non-infected and SBH-infected spheroids at 5, 10, and 15 dpi using CellEvent reagent. **(D)** Quantification of green fluorescence intensity in panel (C), showing increased caspase-3/7 activity in SBH-infected spheroids compared with non-infected controls. **(E)** Representative immunofluorescence images of GFAP staining in non-infected and SBH-infected spheroids at 5, 10, and 15 dpi. Boxed regions are shown at higher magnification. Nuclei were counterstained with DAPI. **(F)** Quantification of GFAP fluorescence intensity in panel (E), demonstrating increased astrogliosis in SBH-infected spheroids, particularly at later time points. **(G)** Representative immunofluorescence images of MAP2 staining in non-infected and SBH-infected spheroids at 10 and 15 dpi. **(H)** Quantification of MAP2 fluorescence intensity in panel (G), showing a significant reduction in neuronal marker expression in SBH-infected spheroids compared with non-infected controls. All scale bars: 100 μm. (For interpretation of the references to color in this figure legend, the reader is referred to the Web version of this article.)

Based on the results from the 2D cell culture system, we attempted to establish a prion-infected, NSC-derived 3D cell culture model using the same differentiation and inoculation procedure as in the 2D system. Over time, the size of the 3D spheroids decreased, with the most dramatic reduction occurring between 1 and 5 dpi, followed by a more gradual decline thereafter (Fig. 2A and B). Interestingly, the shrinkage of prion-infected, NSC-derived 3D spheroids was significantly greater than that of the mock-infected controls and resembled the atrophy observed in brains affected by prion disease.

To investigate the increased apoptosis in prion-infected, NSC-derived 3D spheroids, we measured the green fluorescence intensity of a cleaved substrate, representing the activation of caspase-3/7, using a CellEvent kit. Prion-infected, NSC-derived 3D spheroids exhibited greater green fluorescence intensity than mock-infected controls throughout the experiment, consistent with the observed size reduction (Fig. 2C and D). In addition, gliosis and neuronal degeneration were assessed in prion-infected, NSC-derived 3D spheroids using an immunofluorescence analysis of the astrocyte activation marker GFAP and the neuronal marker MAP2. GFAP expression was significantly elevated in prion-infected spheroids, compared with the mock-infected controls (Fig. 2E and F), whereas MAP2 expression was markedly reduced in prion-infected spheroids, relative to controls (Fig. 2G and H). The increase in GFAP expression indicates astrocyte activation in response to prion infection, and the decrease in MAP2 expression reflects neuronal degeneration, consistent with the observed increase in apoptosis and reduction in spheroid size.

Additional comparison with spheroids treated with brain homogenate from uninfected, age-matched mice showed no detectable differences from the original no-infection control in western blot, GFAP immunofluorescence, or MAP2 immunofluorescence analyses, supporting the use of the no-infection group as the mock control under our experimental conditions (Fig. S1).

### 2.3 PrP^Sc^ accumulation in prion-infected, NSC-derived 3D spheroids

**Fig. 3:**
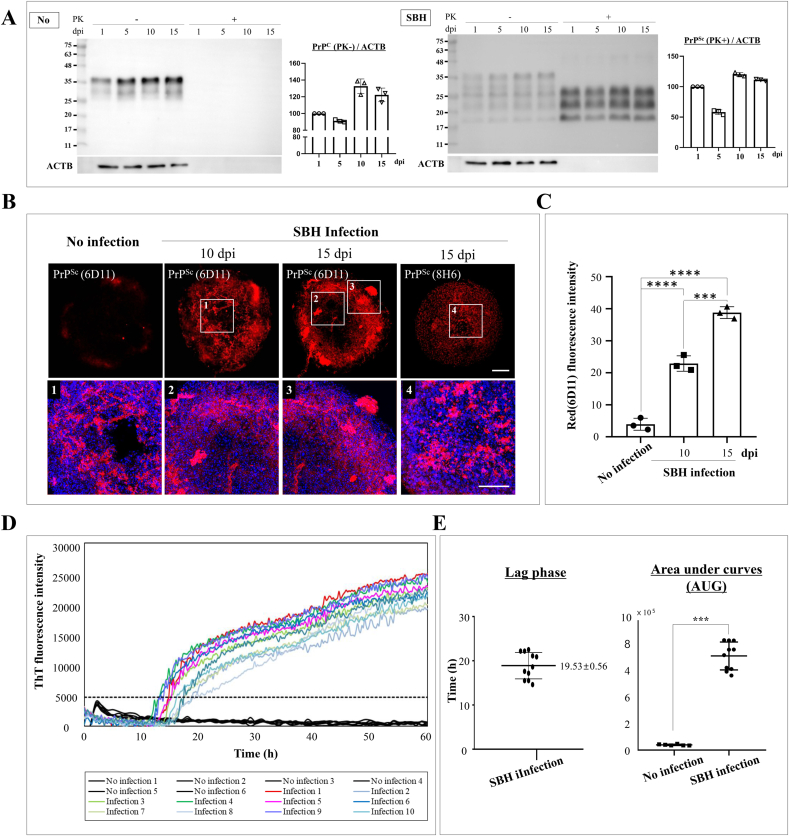
**Detection of PrP^Sc^ accumulation, orthogonal validation, and prion seeding activity in prion-infected NSC-derived 3D spheroid**s. (**A)** Western blot analysis of 3D spheroid lysates with and without proteinase K (PK) treatment. In the no-infection group, PK-resistant PrP^Sc^ was not detected, whereas in the scrapie brain homogenate (SBH)-infected group, PK-resistant PrP^Sc^ was detected at 1, 5, 10, and 15 days post-inoculation (dpi). Quantification of PK-resistant PrP^Sc^ normalized to ACTB is shown on the right. **(B)** Representative immunofluorescence images of PrP^Sc^ staining in non-infected and SBH-infected 3D spheroids. PrP^Sc^ was detected with 6D11 at 10 and 15 dpi, and additional staining with 8H6 at 15 dpi confirmed a comparable PrP^Sc^-positive pattern. Boxed regions are shown at higher magnification. **(C)** Quantification of 6D11 fluorescence intensity, showing increased PrP^Sc^-associated signal in infected spheroids compared with the non-infected group and further elevation at 15 dpi. **(D)** RT-QuIC fluorescence curves obtained from non-infected and infected 3D spheroids. Infected spheroids showed robust ThT fluorescence responses, whereas non-infected spheroids remained below the threshold. **(E)** Quantitative analysis of RT-QuIC responses. Lag-phase analysis of infected spheroids showed no significant differences among individual infected spheroids, indicating relatively uniform seeding kinetics, whereas the area under the curve (AUC) was significantly increased in infected spheroids compared with non-infected controls. All scale bars: 100 μm.

To investigate PrP^Sc^ accumulation in prion-infected, NSC-derived 3D spheroids using orthogonal approaches, we first performed western blot analysis of PK-treated lysates. PK-resistant PrP^Sc^ was detected in infected 3D spheroids, further supporting PrP^Sc^ accumulation in this model (Fig. 3A). We next performed immunofluorescence staining of guanidine thiocyanate-treated 3D spheroids using the mouse monoclonal anti-PrP antibody 6D11 and observed PrP^Sc^-positive signals at 10 and 15 dpi (Fig. 3B). Quantitative analysis of red fluorescence intensity, representing PrP^Sc^, revealed a significant increase in signal with longer propagation time, with a higher signal level at 15 dpi than at 10 dpi (Fig. 3C). To further address the specificity of the immunofluorescence signal, we performed additional PrP^Sc^ staining using a second anti-PrP antibody, 8H6, and observed a comparable staining pattern in prion-infected 3D spheroids (Fig. 3B). Additionally, we assessed prion seeding activity in prion-infected, NSC-derived 3D spheroids by RT-QuIC. Prion-infected 3D spheroids exhibited robust seeding activity, whereas non-infected spheroids showed no detectable signal (Fig. 3D). Each sample was analyzed in triplicate. To further quantify RT-QuIC responses, we analyzed both the area under the fluorescence curve (AUC) and the lag phase (Fig. 3E). Compared with the non-infected spheroids, the prion-infected spheroids showed a significant increase in AUC, indicating enhanced prion seeding activity in the infected group. Lag-phase analysis was used to evaluate consistency among infected spheroids, and no significant differences were observed among individual infected spheroids. This low inter-spheroid variability suggests that RT-QuIC may serve as a useful and reproducible readout for assessing anti-prion drug efficacy.

In addition, RT-QuIC analyses using normal brain homogenate and sick brain homogenate as negative and positive controls, respectively, showed that the fluorescence response of the infected spheroids was comparable to that of the positive control by AUC-based quantification (Fig. S3).

To further address the possibility that the PrP^Sc^ detected in the established 3D spheroid model reflected residual brain homogenate rather than accumulation associated with ongoing infection, we performed two additional analyses. First, immunoblot analysis across early, middle, and late post-inoculation time points showed that the signal was strongest at 1 dpi, consistent with residual inoculum immediately after exposure, whereas the signals detected at 5, 10, and 15 dpi increased progressively thereafter (Fig. 3A). Second, we used PrP^C^-knockout ZH3-derived samples to test whether residual scrapie brain homogenate alone could account for the PK-resistant PrP signal. As shown in Fig. S2A, PrP was detected in N2a and ScN2a cells but not in PrP^C^-knockout ZH3 cells, and PK-resistant PrP^Sc^ was detected only in ScN2a cells after PK digestion. We then inoculated PrP^C^-knockout ZH3-derived spheroids with no brain homogenate, normal brain homogenate (NBH), or scrapie brain homogenate (SBH) and analyzed them with or without PK treatment. No PK-resistant PrP^Sc^ signal was detected in any ZH3 spheroid group, including the SBH-inoculated group (Fig. S2B). If the PrP^Sc^ signal observed in wild-type spheroids had been attributable solely to persistent scrapie brain homogenate, a corresponding PK-resistant band would also be expected in the SBH-inoculated ZH3 spheroids. Together, these findings support the interpretation that residual brain homogenate is largely restricted to the earliest post-inoculation period and that the PrP^Sc^ detected in established wild-type 3D spheroids reflects infection-associated accumulation rather than simple persistence of the original inoculum.

### 2.4 Infectivity of prions propagated in NSC-derived 2D cells and 3D spheroids

**Fig. 4:**
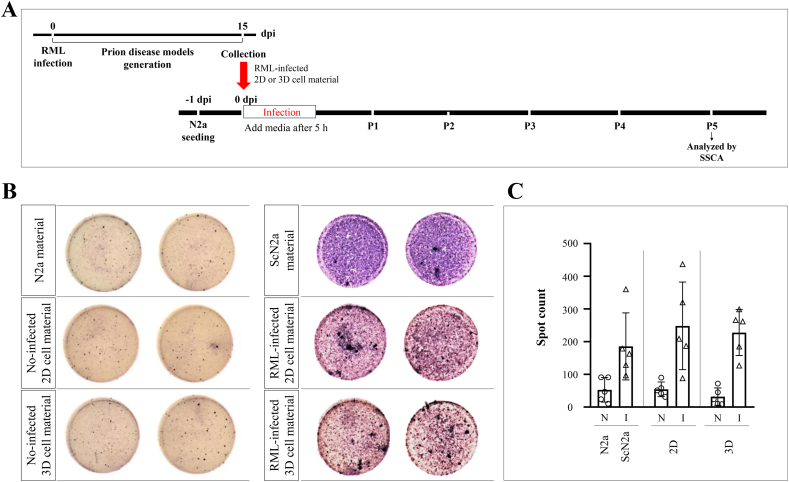
**Standard scrapie cell assay demonstrates infectivity of prions propagated in NSC-derived 2D cells and 3D spheroids.** (A**)** Schematic overview of the standard scrapie cell assay (SSCA). Lysates from prion disease models were collected at 15 dpi and used to inoculate naïve N2a cells. After infection, recipient cells were serially passaged and analyzed by SSCA at passage 5. **(B)** Representative SSCA images of recipient N2a cells inoculated with the indicated materials. N2a material served as a negative control, ScN2a material served as a positive control, and lysates from non-infected or RML-infected 2D cells and 3D spheroids were tested for infectivity. PrP^Sc^-positive spots were detected in cells inoculated with infected 2D and 3D materials, but not in cells inoculated with non-infected controls. **(C)** Quantification of PrP^Sc^-positive spot counts in panel (B), showing retained infectivity of prions propagated in both NSC-derived 2D cells and 3D spheroids.

To evaluate the infectivity of prion-infected, NSC-derived cells, we conducted the standard scrapie cell assay (SSCA) with highly susceptible N2a cells, as illustrated in the timeline in Fig. 4A. The SSCA results obtained with inocula prepared from prion-infected, NSC-derived 2D cells and 3D spheroids clearly demonstrate that the prion-infected cell material induced a substantial number of PrP^Sc^-positive spots in recipient cells, similar to the positive control inoculum of ScN2a material. In contrast, no detectable spots were observed in cells inoculated with non-infected 2D and 3D NSC-derived cells or with the negative control inoculum of N2a material (Fig. 4B and C).

### 2.5 Retention of dimension and PrP^Sc^ loads in cryopreserved prion-infected 3D spheroids

**Fig. 5:**
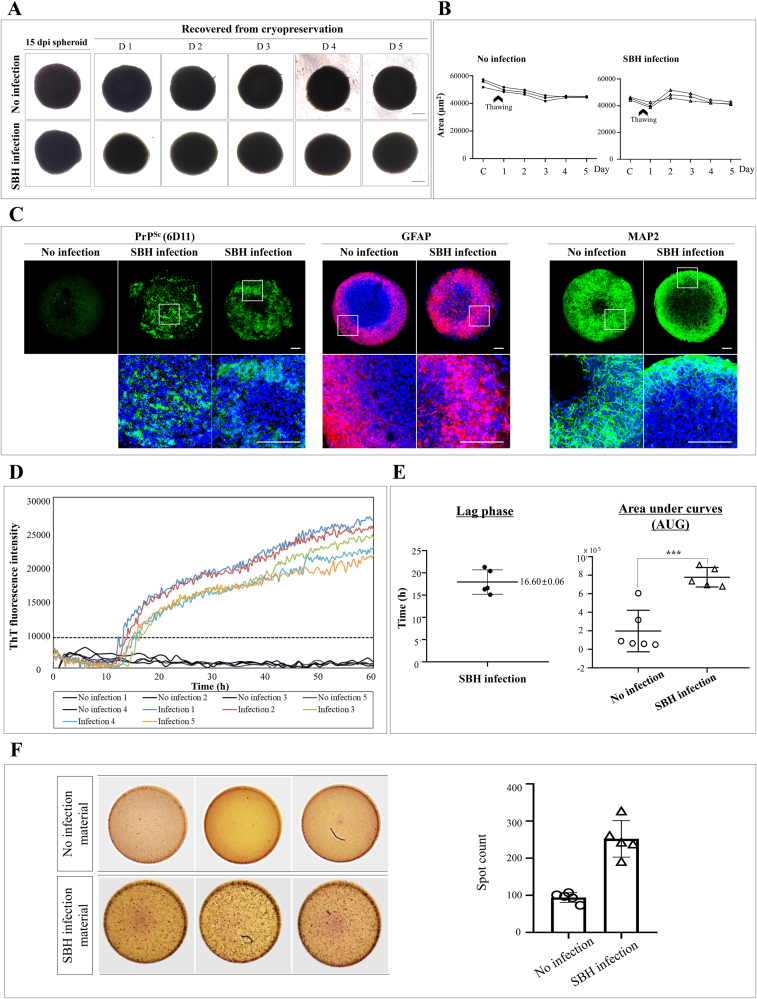
**Preservation of morphology, pathological/cellular features, seeding activity, and infectivity in cryopreserved prion-infected 3D spheroids.** (**A)** Representative bright-field images of 15-dpi spheroids before cryopreservation and during recovery after thawing. Both non-infected and SBH-infected spheroids maintained overall spheroid morphology during the 5-day post-thaw observation period. **(B)** Quantification of spheroid area after thawing, showing no marked reduction in size relative to baseline in either group. **(C)** Representative immunofluorescence images of PrP^Sc^ (6D11), GFAP, and MAP2 in cryopreserved and thawed spheroids. PrP^Sc^ signals and the major neuronal and astrocytic populations were preserved after cryopreservation. Boxed regions are shown at higher magnification. **(D)** RT-QuIC fluorescence curves from cryopreserved spheroids, showing preserved seeding activity in SBH-infected spheroids, whereas non-infected spheroids showed no detectable response. **(E)** Quantitative analysis of RT-QuIC responses after cryopreservation. Lag-phase analysis showed preserved seeding kinetics in infected spheroids, and AUC remained significantly increased compared with the non-infected group. **(F)** SSCA analysis of cryopreserved spheroids after thawing. Representative images and quantification of PrP^Sc^-positive spots showed that lysates from cryopreserved infected spheroids retained biological infectivity compared with non-infected controls. All scale bars: 100 μm.

To assess the stability of the prion-infected, NSC-derived 3D spheroids, we cultured cryopreserved spheroids after thawing and measured their size over a 5-day period. The spheroids remained structurally intact and showed no significant reduction in size, consistent with preserved morphology after thawing (Fig. 5A and B). To evaluate whether PrP^Sc^ was maintained throughout cryopreservation, we measured PrP^Sc^ seeding activity using RT-QuIC. Seeds from cryopreserved prion-infected 3D spheroids showed robust seeding activity with an average lag time of ∼17 h (Fig. 5C and D). The seeding activity of the cryopreserved spheroids was comparable to that of freshly prepared spheroids, as evidenced by their similar lag phases (Fig. 3D).

## 3 Discussion

In this study, we developed a 3D prion model that combines neuronal and astrocytic populations within a spheroid co-culture system to mimic the biochemical and pathological events that occur in affected brains and to provide a platform that is more physiologically relevant than conventional 2D cultures for studying prion pathogenesis. Established prion cell models have mostly relied on 2D cultures of immortalized neuronal cell lines, such as N2a, GT1-7, or CAD5 [[17], [18], [19]], which support PrP^Sc^ accumulation after inoculation with prions. Although these cell lines have proven valuable for studying prion replication and strain characteristics, they cannot replicate the interactions among different neural cell types that occur in the brain. A mixed neuronal and astrocytic cell line, CRBL [20], was reported to exhibit susceptibility to prions, but it has not been explored in a 3D spatial arrangement.

Therefore, the incorporation of astrocytes in our 3D spheroid model is particularly noteworthy, given their essential role in maintaining neuronal function and regulating neuroinflammatory responses. Astrocytes have been shown to influence prion replication and facilitate interactions between neurons and glial cells [21,22], making their presence critical to accurate modeling of prion disease dynamics. By including both neuronal and astrocytic components, our model better reflects neuron–astrocyte interactions than previous 2D models and offers a valuable framework for investigating cell-type-specific responses involved in prion pathogenesis.

Importantly, compared with established 2D prion culture systems, our 3D spheroid model offers several practical and biological advantages. First, the spheroid architecture enables neurons and astrocytes to interact within a spatially organized microenvironment rather than as a flat monolayer, which is expected to better preserve cell–cell communication and local paracrine signaling relevant to CNS pathology [15,23]. Second, because the model is generated from NSCs under defined differentiation conditions in a 96-well format, it combines multicellular complexity with experimental scalability and reproducibility, making it more suitable for comparative and screening studies than animal models and easier to standardize than more complex organoid systems [15,24]. Third, unlike conventional 2D prion-permissive cell lines that are primarily optimized for propagation readouts, this system simultaneously captures prion-associated apoptosis, neuronal marker loss, and astrogliosis in the same culture context, thereby providing a broader phenotypic window for mechanistic investigation and therapeutic evaluation [19,21,25]. Accordingly, we consider this model to be a complementary intermediate platform that bridges simple 2D cultures and more complex in vivo or organoid-based systems for prion research.

Our NSC-derived 3D spheroid model not only supports robust PrP^Sc^ formation but also retains prion infectivity. Lysates from infected spheroids successfully induced *de novo* PrP^Sc^ formation in naïve N2a cells after serial passages, as confirmed by spot quantification using the SSCA. To our knowledge, this is the first demonstration of sustained infectivity and prion transmission from a 3D NSC-based prion model, representing a key advance over other systems.

Beyond modeling prion propagation, our 3D spheroid system exhibited several quantifiable and reproducible pathological phenotypes, making it well suited for pharmacological testing. Upon prion infection, the spheroids showed shrinkage in volume (resembling the atrophy of an affected brain), elevated caspase-3/7 activity (representing apoptosis), a substantial decrease in MAP2 expression (representing neuronal degeneration), a significant increase in GFAP expression (representing astrogliosis), and a clear, time-dependent increase in PrP^Sc^ accumulation (representing the deposition of PrP^Sc^ in an affected brain). Because the molecular, cellular, and tissue-level events observed in our 3D spheroid system faithfully recapitulate the biochemical and pathological changes found in affected brains, these measurable markers provide a useful tool for estimating the efficacy of potential anti-prion agents. Notably, RT-QuIC analysis revealed reproducible seeding activity in prion-infected spheroids. The area under the fluorescence curve (AUC) was used to confirm infection status and showed a significant increase in infected spheroids compared with non-infected controls. By contrast, lag-phase analysis was applied to individual infected spheroids and revealed no significant differences among them, indicating that prion seeding activity was relatively uniform across infected spheroids. These findings indicate low variability in seeding kinetics within the infected group and support the potential utility of RT-QuIC as a sensitive readout for assessing relative anti-prion drug efficacy.

No previous studies have examined the cryopreservation of prion-infected 3D neuronal culture models. A recent review of prion cell culture models highlighted several 3D systems that replicate prion propagation and toxicity, including spheroids derived from CAD5 cells [25] and cerebral organoids from human induced pluripotent stem cells [24]. However, those models did not evaluate whether their prion-infected cultures retained pathogenic properties after cryopreservation. In this study, we demonstrated that our prion-infected, NSC-derived 3D spheroids preserved both their structural integrity and prion seeding activity after cryopreservation, as confirmed by spheroid morphology and RT-QuIC analysis. These results highlight the scalability and practical utility of our model for reproducible experimentation, storage, and inter-laboratory distribution in prion research and drug screening contexts.

In summary, we have presented a 3D NSC-derived prion model that captures critical aspects of prion pathology, including PrP^Sc^ accumulation, sustained seeding activity, and prion infectivity. The model demonstrates strong utility for pharmacological evaluation and retains pathological, cellular, and structural features after cryopreservation, facilitating storage and inter-laboratory applications. Taken together, these features support our model as a versatile and experimentally tractable system for mechanistic studies and drug screening in prion disease.

## 4 STAR★Methods

**Table:** Key resources table

REAGENT or RESOURCE	SOURCE	IDENTIFIER
**Antibodies**		
PrP (for western blot and Immunofluorescence)	BioLegend	Cat#808003; RRID: AB_2564737
PrP (for Immunofluorescence)	Sigma	P0110,
MAP2 (for Immunofluorescence)	Thermo Fisher Scientific	Cat#13-1500; RRID: AB_2533001
SOX2 (for Immunofluorescence)	R&D Systems	Cat#AF2018; RRID: AB_355110
Nestin (for Immunofluorescence)	R&D Systems	Cat#MAB1259; RRID: AB_2251304
Tuj-1 (for Immunofluorescence)	R&D Systems	Cat#MAB1195; RRID: AB_357520
GFAP (for Immunofluorescence)	Dako	Cat#Z0334; RRID: AB_10013382
**Chemicals, peptides, and recombinant proteins**		
DMEM	GIBCO	Cat#12491015
FBS	GIBCO	Cat#A5256701
Penicillin/streptomycin	GIBCO	Cat#15140122
Poly-L-ornithine	Sigma-Aldrich	Cat#27378-49-0
Fibronectin	Sigma-Aldrich	Cat#CLS354008
DMEM/F12	GIBCO	Cat#12634010
N-2 supplement	GIBCO	Cat#17502001
D-glucose	GIBCO	Cat#A2494001
Non-essential amino acids	GIBCO	Cat#11140050
β-mercaptoethanol	GIBCO	Cat#21985023
Epidermal growth factor	Thermo Fisher Scientific	Cat#PHG0314
Basic fibroblast growth factor	Peprotech	Cat#100-18B
PLO/laminin	Sigma-Aldrich	Cat#LPLO001
B-27^TM^ plus supplement	Sigma-Aldrich	Cat#A3582801
Nerve growth factor	GIBCO	Cat#PHG0126
Proteinase K	Roche	Cat#48686700
Phenylmethylsulfonyl fluoride	Merck Millipore	Cat#52332
4′,6-diamidino-2-phenylindole	Thermo Fisher Scientific	Cat#202710100
Spectra/Por® 1 RC dialysis tubing	Repligen	Cat#08670B
Histrap™ HP	Cytiva	Cat#28411001
HiTrap™ desalting	Cytiva	Cat#17140801
high-protein-binding Immobilon-P membrane	Merck Millipore	Cat#IPVH00010
SuperBlock solution	Thermo Fisher Scientific	Cat#37535
**Experimental models: Cell lines**		
N2a cell line	Mouse	CCL-131
**Experimental models: Organisms/strains**		
Mouse neural stem cell	The Jackson Laboratory	FVB/NJ mice; 001800 ZH3 mice (PrP knockout mice); N/A
**Biological samples**		
Brain homogenate	Terminally ill RML prion-infected mice	N/A
**Software and algorithms**		
ÄKTA go™ system	Cytiva	https://www.cytivalifesciences.co.kr/model/akta-go/
FLUOstar Omega fluorescence plate reader	BMGLabtech	https://www.bmglabtech.com/ko/fluostar-omega/
S6 Universal M2 ELISPOT reader	ImmunoSpot	https://immunospot.com/immunospot-s6-universal-m2.html
ImmunoSpot software	ImmunoSpot	https://immunospot.com/immunospot-s6-universal-m2.html
GraphPad Prism	San Diego	https://www.graphpad.com/features
Sigma Plot	Palo Alto	https://sigmasw.co.kr/product/product-01/?ckattempt=1

### 4.1 Isolation and differentiation of neural stem cells

FVB/NJ mice were purchased from Jackson Laboratory (Bar Harbor, ME, USA). NSCs were isolated from the embryonic forebrain at E12.5, based on a previously described protocol [26]. The isolated cells were dissociated in Dulbecco's Modified Eagle Medium (DMEM; GIBCO, Carlsbad, CA, USA) supplemented with 10% fetal bovine serum (FBS; GIBCO) and 0.1 mg/mL penicillin/streptomycin (GIBCO) and then plated onto tissue culture dishes coated with poly-L-ornithine (PLO; 15 μg/mL, Sigma-Aldrich, St. Louis, MO, USA) and fibronectin (1 μg/mL, Sigma-Aldrich). On the following day, the culture medium was replaced with NSC medium consisting of DMEM/F12 supplemented with 100× N2 (GIBCO), 300 mM D-glucose (GIBCO), 200 mM L-glutamine (GIBCO), 100 U/mL penicillin (GIBCO), 100 μg/mL streptomycin (GIBCO), 0.1 mM non-essential amino acids (GIBCO), 0.1 mM β-mercaptoethanol (GIBCO), 10 ng/mL epidermal growth factor (EGF; Thermo Fisher Scientific, Waltham, MA, USA), and 10 ng/mL basic fibroblast growth factor (Peprotech, Rocky Hill, NJ, USA). The NSCs were maintained at 37°C in a humidified incubator with 5% CO_2_ and passaged every 3 days.

To differentiate the NSCs into mature neurons, they were plated directly onto PLO/laminin-coated dishes (5 μg/mL, Sigma-Aldrich). The differentiation protocol was adapted from a previously described method [27] and involved the withdrawal of EGF and a gradual reduction of fibroblast growth factor-2 (FGF-2; Thermo Fisher Scientific). Briefly, the differentiation medium consisted of DMEM/F12 supplemented with 200× N2, 100× B27 (Sigma-Aldrich), 100 U/mL penicillin, 100 μg/mL streptomycin, 0.1 mM non-essential amino acids, 0.1 mM β-mercaptoethanol, 3 mM D-glucose, and 2 mM L-glutamine. For the first 3 days, 20 ng/mL nerve growth factor (NGF; GIBCO) and 10 ng/mL FGF-2 were added (neural medium A). After that, the medium was supplemented with 30 ng/mL NGF and 6.7 ng/mL FGF-2 for an additional 14 days (neural medium B).

### 4.2 Prion infection of NSC-derived 2D and 3D cells

To establish prion infection in NSC-derived 2D monolayer and 3D spheroid cultures, we used a previously described protocol with minor modifications [27]. NSCs were pre-cultured in neural medium A for 3 days to support early differentiation. On day 3, the cells were inoculated with 10% (w/v) brain homogenate derived from terminally ill RML prion-infected mice diluted in neural medium B. After 24 h, the medium was replaced with fresh neural medium B, and the cultures were maintained for an additional 15 days with daily medium changes. Samples were collected at 5, 10, and 15 dpi for analysis. Both the 2D cultures and 3D spheroids underwent immunofluorescence, western blotting, and RT-QuIC assays to monitor PrP^Sc^ accumulation and seeding activity.

### 4.3 3D spheroid morphology and size

To evaluate the morphological changes and growth dynamics of the 3D spheroids over time, bright-field images were acquired at defined intervals (0, 1, 5, 10, and 15 dpi), as described previously [23]. Spheroids were cultured in ultra-low attachment 96-well round-bottom plates (Corning, NY, USA) and imaged using an inverted phase-contrast microscope (Olympus, Tokyo, Japan) at 4× magnification. For each point, multiple spheroids (n = 3 per group) were imaged, and the projected 2D area was measured using ImageJ software (National Institutes of Health, Bethesda, MD, USA). The area of each spheroid was quantified by outlining the perimeter manually, and the results are presented as the mean ± SEM.

### 4.4 Western blot analysis of PrP^C^ and PrP^Sc^

PrP^C^ and PrP^Sc^ in the cultured cells and spheroids were detected as described previously, with minor modifications [28]. To prepare cell lysates, 2D cells in 60 mm culture dishes or eight 3D spheroids were lysed in buffer containing 50 mM Tris-HCl (pH 7.4), 150 mM NaCl, 0.5% Nonidet P-40, and 0.5% sodium deoxycholate (Sigma-Aldrich). The lysates were subjected to 20 cycles of sonication for 30 s followed by incubation on ice for 30 s. The samples were then centrifuged at 12,000 × g for 15 min at 4°C, and the supernatants were collected. Protein concentrations were measured using the bicinchoninic acid assay (Thermo Fisher Scientific).

To detect PrP^Sc^, 0.2 mg of total protein were incubated with 20 μg/mL PK (Roche, Basel, Switzerland) at 37°C for 1 h. The reaction was stopped by adding 2 mM phenylmethylsulfonyl fluoride (PMSF; Merck Millipore, Burlington, MA, USA), and then centrifuged at 16,000 × *g* for 75 min at 4°C. The resulting pellets were resuspended in 4× sample loading buffer (Thermo Fisher Scientific) and heated at 100°C for 10 min. To detect PrP^C^ and β-actin, 20 μg of total protein were used without PK digestion.

Equal amounts of protein were loaded onto 15% sodium dodecyl sulfate (SDS)-polyacrylamide gels and separated by electrophoresis. Proteins were transferred to polyvinylidene difluoride membranes (Merck Millipore), incubated with primary antibodies against the target proteins, and subsequently incubated with horseradish peroxidase-conjugated secondary antibodies. Protein bands were visualized using WesternBright™ ECL (Advansta, San Jose, CA, USA) and imaged with a G:Box Chemi XR5 system (Syngene, Cambridge, UK). The primary antibodies used for western blotting were mouse monoclonal anti-PrP antibody 6D11 (1:3000, BioLegend, San Diego, CA, USA) and anti-β-actin.

### 4.5 Immunofluorescence

The immunofluorescence study of the cultured cells and spheroids was performed as described previously [23]. Two-dimensional cells or 3D spheroids were fixed with 3.7% formalin (Duksan General Science, Seoul, Korea) for 30 min at room temperature and then washed with phosphate buffered saline (PBS). The fixed 2D cells were incubated in a blocking buffer (10% bovine serum albumin and 0.3% Triton X-100 in PBS) for 30 min at room temperature. Thereafter, the samples were incubated overnight with primary antibodies at 4°C and then with the appropriate fluorescent probe–conjugated secondary antibodies for 1 h at room temperature, protected from light.

To perform immunofluorescence staining of fixed 3D spheroids, permeabilization was carried out using 0.2% Triton X-100 (Sigma-Aldrich) overnight at 4°C. To detect PrP^Sc^, the spheroids were treated with 5 M guanidine thiocyanate (Sigma-Aldrich) for 15 min at room temperature and then blocked with 2% bovine serum albumin (Sigma-Aldrich) overnight at 4°C. The samples were then incubated overnight at 4°C with the primary antibodies, followed by incubation with the appropriate fluorescent probe-conjugated secondary antibodies overnight at 4°C in the dark. After completion of both the primary and secondary antibody incubations, nuclei were counterstained with 4′,6-diamidino-2-phenylindole (DAPI; Thermo Fisher Scientific). Images were captured using a confocal microscope (FV3000, Olympus, Tokyo, Japan). The primary antibodies used in this study were anti-PrP antibody 6D11 (1:200, BioLegend), anti-PrP antibody 8H6 (1:200, Sigma), anti-MAP2 antibody (1:100, Thermo Fisher Scientific), anti-SOX2 antibody (1:200, R&D Systems, Minneapolis, MN, USA), anti-Nestin antibody (1:200, R&D Systems), anti-Tuj-1 antibody (1:200, R&D Systems), and anti-GFAP antibody (1:200, Dako, Agilent Technologies, Carpinteria, CA, USA).

### 4.6 Purification of recombinant PrP

Recombinant PrP was purified as previously described [29]. Briefly, cDNA encoding His-tagged mouse PrP (residues 23–231) was cloned into the pET100/D-TOPO vector (Invitrogen, Carlsbad, CA, USA). The construct was transformed into *E. coli* BL21 Star (DE3) cells (Invitrogen), and protein expression was induced with 1 mM isopropyl β-D-1-thiogalactopyranoside (iNtRON, Seongnam-si, Gyeonggi-do, Korea), followed by incubation for 16 h at 37°C. The cells were harvested and lysed by sonication in a lysis buffer composed of 10% sucrose (Sigma-Aldrich), 0.1 M Tris-HCl (Sigma-Aldrich), 50 mM EDTA (pH 8.0; Invitrogen), 0.2 M NaCl (pH 7.9; Sigma-Aldrich), and 1 mM PMSF (Merck Millipore). The resulting inclusion bodies were solubilized in solubilization buffer (8 M urea (Samchun, Seoul, Korea), 10 mM glycine (pH 10.6; Sigma-Aldrich)) and refolded in refolding buffer (0.6 M urea, 10 mM glycine, pH 10.6). During refolding, the proteins were dialyzed in Spectra/Por® 1 RC dialysis tubing (6–8 kDa MWCO; Repligen) with gentle stirring at room temperature. The dialysis buffer was stepwise exchanged every 2 h from 4 M urea → 2 M urea → 1 M urea (in 10 mM glycine, pH 10.6), followed by overnight dialysis against the urea-free buffer at room temperature. His-tagged recombinant PrP was purified using immobilized metal affinity chromatography with a Histrap™ HP (Cytiva, Uppsala, Sweden), followed by desalting using HiTrap™ desalting (Cytiva), both performed on an ÄKTA go™ system (Cytiva). The purified recombinant protein was verified by Coomassie blue staining, and aliquots were stored at −80°C until use.

### 4.7 Real-time quaking-induced conversion

RT-QuIC was performed as previously described [30]. Briefly, 10 μg of recombinant PrP and prion seeds (5 × 10^-5 diluted cell lysate or 5 × 10^-4 diluted 3D cell lysate) were mixed in 100 μL of RT-QuIC reaction buffer containing 0.5 M NaCl, 10 μM EDTA, 10 mM phosphate buffer (pH 7.4; Sigma-Aldrich), 10 μM ThT (Sigma-Aldrich), 0.002% SDS (Sigma-Aldrich), 0.05% trifluoroacetic acid (Sigma-Aldrich), and 2% acetonitrile (Sigma-Aldrich). All reagents were passed through a 0.2 μm syringe filter (Sartorius, Göttingen, Germany) before use. Prion seeds were derived from cell lysates of either 2D- or 3D-cultured cells, with or without prion infection. Reaction mixtures were dispensed into black, clear-bottom, 96-well non-treated polystyrene microplates (Corning, Corning, NY, USA), sealed with adhesive film (USA Scientific, Ocala, FL, USA), and incubated at 42°C for 60 h with orbital shaking at 700 rpm in a FLUOstar Omega fluorescence plate reader (BMGLabtech, Ortenberg, Germany). ThT fluorescence was monitored every 15 min in bottom-read mode (excitation, 450 nm; emission, 480 nm) to assess amyloid formation and prion seeding activity. Each biological sample was analyzed in three technical replicate wells.

### 4.8 Standard scrapie cell assay

To assess the infectivity of prion-infected 3D spheroids, we used the SSCA, a well-established in vitro method for quantifying prion infectivity in cell cultures, following procedures described previously [30]. NSC-derived 3D spheroids were harvested 15 days after inoculation with RML prions and homogenized in sterile PBS using mechanical trituration with 10 on/off cycles of 10 s each at 30% amplitude (Qsonica LLC, Newtown, CT, USA) followed by brief sonication on ice. The homogenates were clarified by centrifugation at 2000 × g for 5 min at 4°C, and the supernatant was used as the inoculum for the following SSCA. N2a cells, which are highly susceptible to RML prion infection, were seeded at 2 × 10^4^ cells/well in 24-well plates and exposed to the inoculum (1:10 dilution in DMEM) for 5 h. Then, the cells were thoroughly washed and maintained in standard growth medium (DMEM with 10% FBS, 1% GlutaMAX (GIBCO), and 1% penicillin/streptomycin). The cells were grown for several passages with fresh culture medium every 3–4 days without additional exposure to infectious material. The cells at the fifth passage (2.5 × 10^4^ cells) were seeded onto an activated opaque 96-well plate with a 0.45-μm hydrophobic, high-protein-binding Immobilon-P membrane (Merck Millipore, Darmstadt, Germany). The cells were then fixed with 4% paraformaldehyde and subjected to PK (5 μg/mL) digestion for 90 min at 37°C to remove endogenous PrP^C^. The reaction was stopped by adding 2 mM PMSF (Merck Millipore) and PBS, followed by shaking for 20 min. The membranes were soaked in denaturation buffer (3 M guanidine thiocyanate in 10 mM Tris-HCl) for 10 min and rinsed five times with PBS. Blocking was performed using SuperBlock solution (Thermo Fisher Scientific, Waltham, MA, USA). To detect PrP^Sc^, the membranes were incubated with mouse monoclonal anti-PrP antibody 6D11 (1:10,000; BioLegend, San Diego, CA, USA), followed by incubation with alkaline phosphatase–conjugated mouse IgG (1:5000; Abcam, Cambridge, UK). Prion-infected cells were visualized and quantified using an S6 Universal M2 ELISPOT reader (ImmunoSpot, Shaker Heights, OH, USA) and analyzed with ImmunoSpot software (ImmunoSpot, Shaker Heights, OH, USA) after color development using nitro-blue tetrazolium chloride/5-bromo-4-chloro-3ʹ-indolyl phosphate *p*-toluidine salt substrate.

### 4.9 Cryopreservation and thawing of prion-infected 3D spheroids

Prion-infected 3D spheroids collected 15 dpi were cryopreserved in 500 μL of freezing medium consisting of 50% neural medium B, 40% FBS, and 10% dimethyl sulfoxide (DMSO). The spheroids were gradually cooled using a controlled-rate freezing container (Mr. Frosty™, Thermo Fisher) and stored at −80°C overnight before they were transferred to liquid nitrogen for long-term storage. For thawing, the cryovials were rapidly warmed in a 37°C water bath, and the spheroids were immediately transferred into pre-warmed culture medium to dilute residual DMSO and then maintained in ultra-low attachment plates for further analysis.

### 4.10 Statistical analysis

Data were analyzed using two-way ANOVAs followed by Tukey's post-hoc tests or one-sample t-tests in GraphPad Prism (version 5.0; San Diego, CA, USA). Statistical significance was accepted at the 95% probability level. Data are presented as the mean ± standard error of the mean. Sigmoidal plot analysis was performed to assess the seeding efficiency and kinetics of prion propagation using the logistic curve-fitting function of Sigma Plot (version 12, Palo Alto, CA, USA). After fitting the fluorescence data with a logistic model, the lag phase was estimated using an empirical formula derived from the fitted parameters:

Lag phase = x_0_ ∗ ((|*p*|-1)/(|*p*|+1))^1/|*p*|^

where x0 represents the midpoint (time to 50% aggregation) and p is the slope parameter obtained from nonlinear regression. This approximation has been used in previous studies modeling sigmoidal kinetics of protein aggregation and microbial growth [31]. To quantify the total ThT fluorescence signal, the area under the curve was calculated using the trapezoidal rule in Microsoft Excel (version 2016, Microsoft Korea, Seoul, Korea). Fluorescence intensities were measured every 15 min (0.25 h), and the area between two adjacent time points was computed as [(F_n_ + F_n+1_)/2] × 0.25. A reference line was added to facilitate visual comparison of the average lag phase.

## Lead contact

Requests for further information and resources should be directed to and will be fulfilled by the lead contact, Hyun Jung Park (pphj0105@gmail.com).

## CRediT authorship contribution statement

**Hyun Jung Park:** Conceptualization, Data curation, Formal analysis, Funding acquisition, Investigation, Methodology, Project administration, Resources, Software, Supervision, Validation, Visualization, Writing – original draft, Writing – review & editing. **Hyeri Kim:** Formal analysis, Methodology. **Sanghoon Byun:** Formal analysis, Investigation, Methodology, Software. **Chongsuk Ryou:** Conceptualization, Funding acquisition, Methodology, Project administration, Supervision, Validation, Writing – review & editing.

## Declaration of competing interest

The authors declare that they have no known competing financial interests or personal relationships that could have appeared to influence the work reported in this paper.

## Data Availability

Data will be made available on request.

## References

[1] Prusiner S.B. (1998). Prions. Proc. Natl. Acad. Sci. U. S. A.

[2] Schonberger L.B. (1998). New variant Creutzfeldt-Jakob disease and bovine spongiform encephalopathy. Infect. Dis. Clin..

[3] Tuite M.F., Serio T.R. (2010). The prion hypothesis: from biological anomaly to basic regulatory mechanism. Nat. Rev. Mol. Cell Biol..

[4] Ryou C., Mays C.E. (2008). Prion propagation in vitro: are we there yet?. Int. J. Med. Sci..

[5] Safar J. (1998). Eight prion strains have PrP(Sc) molecules with different conformations. Nat. Med..

[6] Aguzzi A. (2008). Molecular mechanisms of prion pathogenesis. Annu. Rev. Pathol..

[7] Trevitt C.R., Collinge J. (2006). A systematic review of prion therapeutics in experimental models. Brain.

[8] Fernandez-Borges N. (2013). Animal models for testing anti-prion drugs. Curr. Top. Med. Chem..

[9] Colini Baldeschi A. (2020). Novel regulators of PrP(C) expression as potential therapeutic targets in prion diseases. Expert Opin. Ther. Targets.

[10] Kumar R. (2008). Cholesterol transporter ATP-binding cassette A1 (ABCA1) is elevated in prion disease and affects PrPC and PrPSc concentrations in cultured cells. J. Gen. Virol..

[11] Milhavet O. (2006). Neural stem cell model for prion propagation. Stem Cell..

[12] Giri R.K. (2006). Prion infection of mouse neurospheres. Proc. Natl. Acad. Sci. U. S. A.

[13] Herva M.E. (2010). Prion infection of differentiated neurospheres. J. Neurosci. Methods.

[14] Iwamaru Y. (2013). Prion replication elicits cytopathic changes in differentiated neurosphere cultures. J. Virol..

[15] Lee C.J. (2025). Advanced animal replacement testing strategies using stem cell and organoids. Int. J. Stem Cells.

[16] Bueler H. (1992). Normal development and behaviour of mice lacking the neuronal cell-surface PrP protein. Nature.

[17] Klohn P.C. (2003). A quantitative, highly sensitive cell-based infectivity assay for mouse scrapie prions. Proc. Natl. Acad. Sci. U. S. A.

[18] Mahal S.P. (2007). Prion strain discrimination in cell culture: the cell panel assay. Proc. Natl. Acad. Sci. U. S. A.

[19] Milhavet O. (2000). Prion infection impairs the cellular response to oxidative stress. Proc. Natl. Acad. Sci. U. S. A..

[20] Mays C.E. (2008). CRBL cells: establishment, characterization and susceptibility to prion infection. Brain Res..

[21] Hartmann K. (2019). Complement 3(+)-astrocytes are highly abundant in prion diseases, but their abolishment led to an accelerated disease course and early dysregulation of microglia. Acta Neuropathol. Commun..

[22] Moser M. (1995). Developmental expression of the prion protein gene in glial cells. Neuron.

[23] Park H. (2023). A three-dimensional spheroid co-culture system of neurons and astrocytes derived from Alzheimer's disease patients for drug efficacy testing. Cell Prolif..

[24] Groveman B.R. (2021). Cerebral organoids as a new model for prion disease. PLoS Pathog..

[25] Fremuntova Z. (2024). Simple 3D spheroid cell culture model for studies of prion infection. Eur. J. Neurosci..

[26] Currle D.S. (2007). Culture of mouse neural stem cell precursors. J. Vis. Exp..

[27] Li E. (2020). Neural stem cells derived from the developing forebrain of YAC128 mice exhibit pathological features of Huntington's disease. Cell Prolif..

[28] Kim D.H. (2023). Synthesis and anti-prion aggregation activity of acylthiosemicarbazide analogues. J. Enzym. Inhib. Med. Chem..

[29] Hwang H.G. (2018). High-level production of high-purity human and murine recombinant prion proteins functionally compatible to in vitro seeding assay. J. Microbiol. Biotechnol..

[30] Lee S. (2022). The effect of Curcuma phaeocaulis Valeton (Zingiberaceae) extract on prion propagation in cell-based and animal models. Int. J. Mol. Sci..

[31] Zwietering M.H. (1990). Modeling of the bacterial growth curve. Appl. Environ. Microbiol..

