# Supplementary material for: A neural stem cell–derived 3D spheroid model that recapitulates prion infection and pathology
Source: Mater Today Bio. 2026 Jun 22;39:103378. doi: 10.1016/j.mtbio.2026.103378 (PMC13315006; doi:10.1016/j.mtbio.2026.103378)
Supplement: Multimedia component 1 [file mmc1.docx]

**Supplementary Information**

**A neural stem cell–derived 3D spheroid model that recapitulates prion infection and pathology**

**Hyun Jung Park^1,2,*^, Hyeri Kim^1^, Sanghoon Byun^1^, Chongsuk Ryou^1,*^**

^1^Department of Pharmacy, College of Pharmacy, and Institute of Pharmaceutical Science & Technology, Hanyang University ERICA, 55 Hanyangdaehak-ro, Ansan, Gyeonggi-do, 15588, Republic of Korea

^2^Department of Health Science and Technology, Samsung Advanced Institute of Health Sciences and Technology (SAIHST), Sungkyunkwan University

*Co-corresponding authors:

Hyun Jung Park, Ph.D. E-mail: [pphj0105@gmail.com](mailto:pphj0105@gmail.com)

81 Irwon-ro, Gangnam-gu, Seoul, 06351, Sungkyunkwan University, Republic of Korea

Chongsuk Ryou, Ph.D. E-mail: [cryou2@hanyang.ac.kr](mailto:cryou2@hanyang.ac.kr)

55 Hanyangdaehak-ro, Sangnok-gu, Ansan, Gyeonggi-do, 15588, Republic of Korea

**Contents**

Figure S1. Comparison of the no-infection control and the normal brain homogenate-treated control in NSC-derived 3D spheroids.

Figure S2. Additional western blot controls for PrP detection and PK resistance.

Figure S3. RT-QuIC positive and negative controls and AUC-based comparison with infected spheroids.


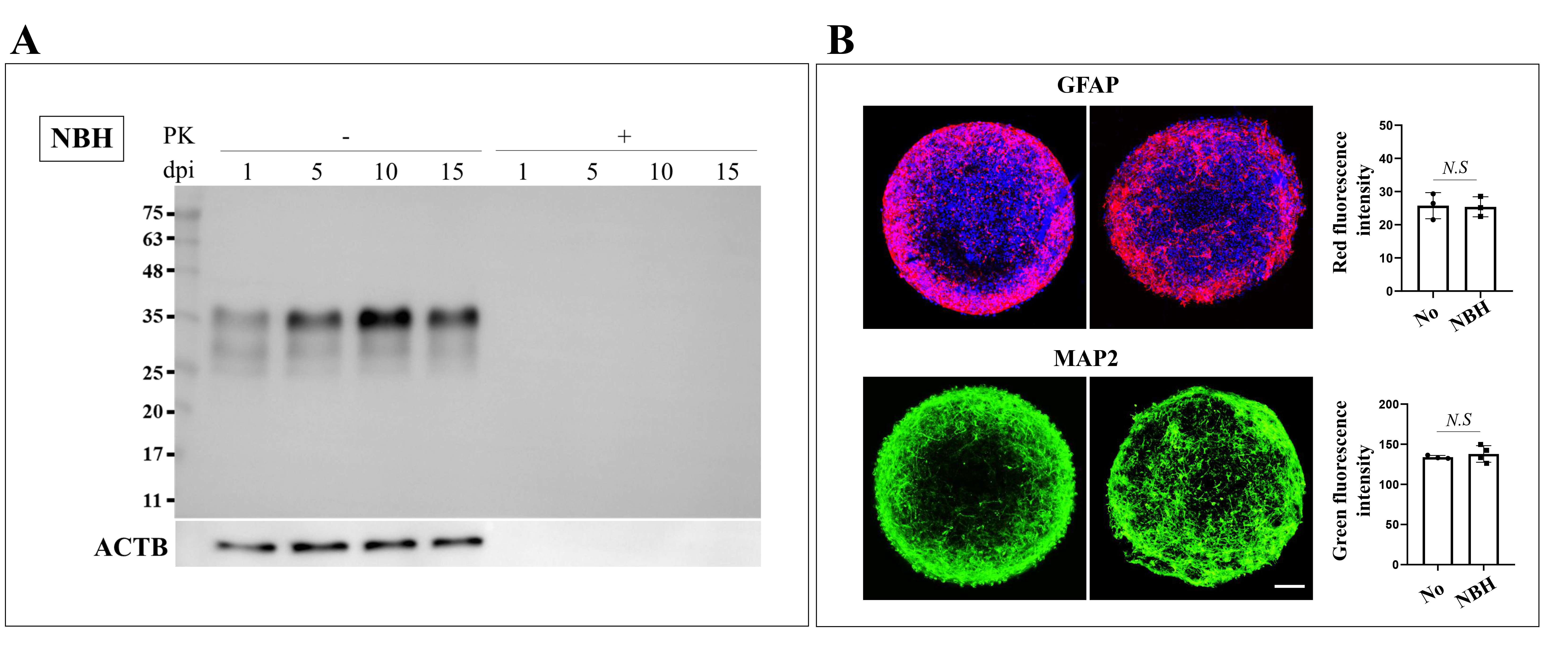


**Figure S1. Comparison of the no-infection control and the normal brain homogenate-treated control in NSC-derived 3D spheroids.**
**(A)** Western blot analysis of spheroids treated with normal brain homogenate (NBH) at the indicated time points with and without proteinase K (PK) treatment. No PK-resistant PrPSc signal was detected after PK treatment. ACTB was used as a loading control.
**(B)** Representative immunofluorescence images and quantification of GFAP and MAP2 in no-infection and NBH-treated spheroids. No significant differences were observed between the two groups in GFAP or MAP2 fluorescence intensity. All scale bars: 100 μm.

**Figure S2. Additional western blot controls for PrP detection and PK resistance.**


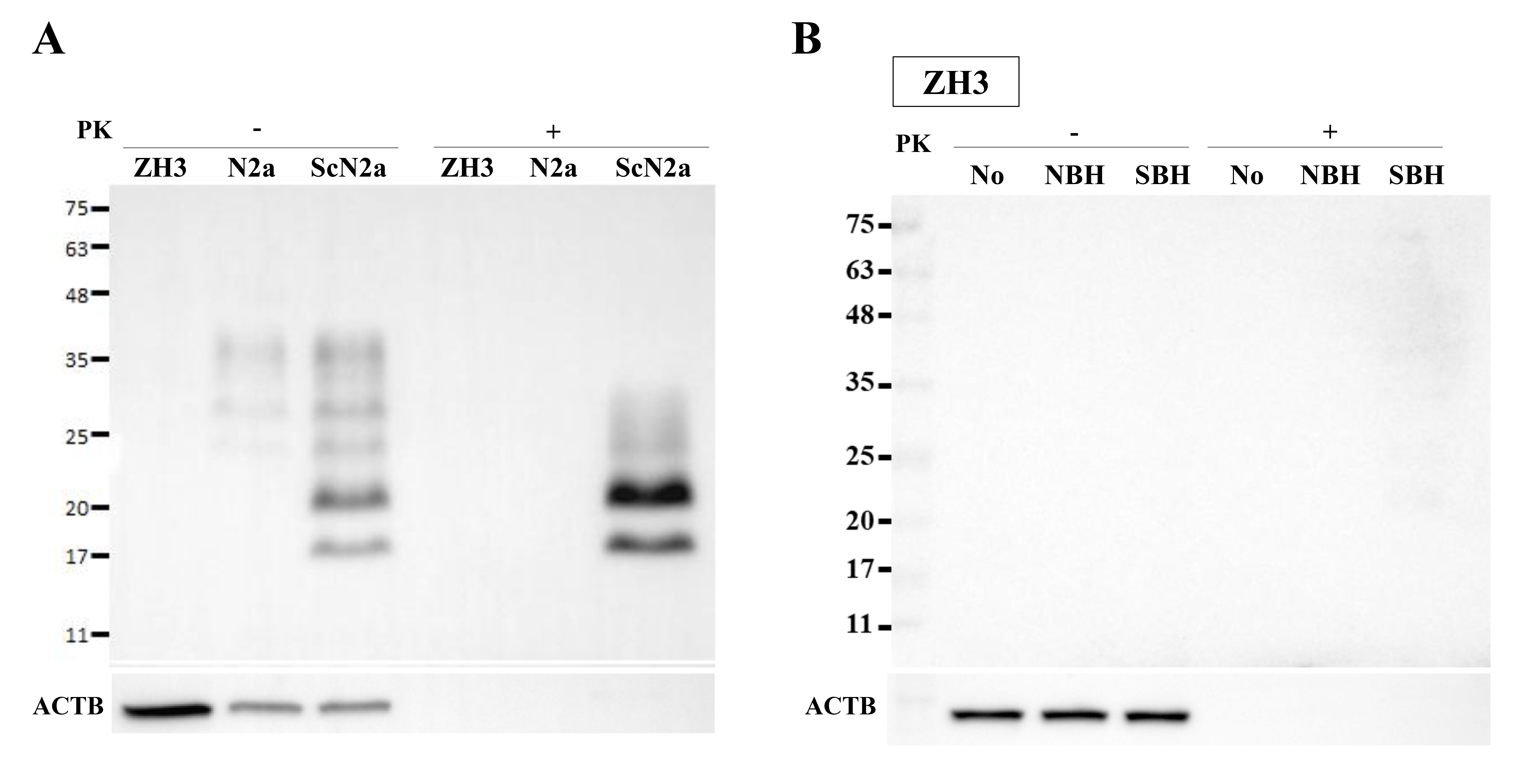


**(A) Western blot analysis of ZH3, N2a, and ScN2a cell lysates in the absence or presence of proteinase K (PK) treatment. PrP signals were detected in N2a and ScN2a cells without PK treatment, whereas no PrP signal was detected in PrP^C^-knockout ZH3 cells. After PK treatment, PK-resistant PrP^Sc^ was detected only in ScN2a cells. ACTB was used as a loading control for non-PK-treated samples.**

**(B) Western blot analysis of PrP^C^-knockout ZH3-derived spheroids inoculated with no brain homogenate (No), normal brain homogenate (NBH), or scrapie brain homogenate (SBH), with or without PK treatment. No PK-resistant PrP^Sc^ signal was detected in any ZH3-derived spheroid group after PK digestion, including the SBH-inoculated group. ACTB was used as a loading control for non-PK-treated samples.**


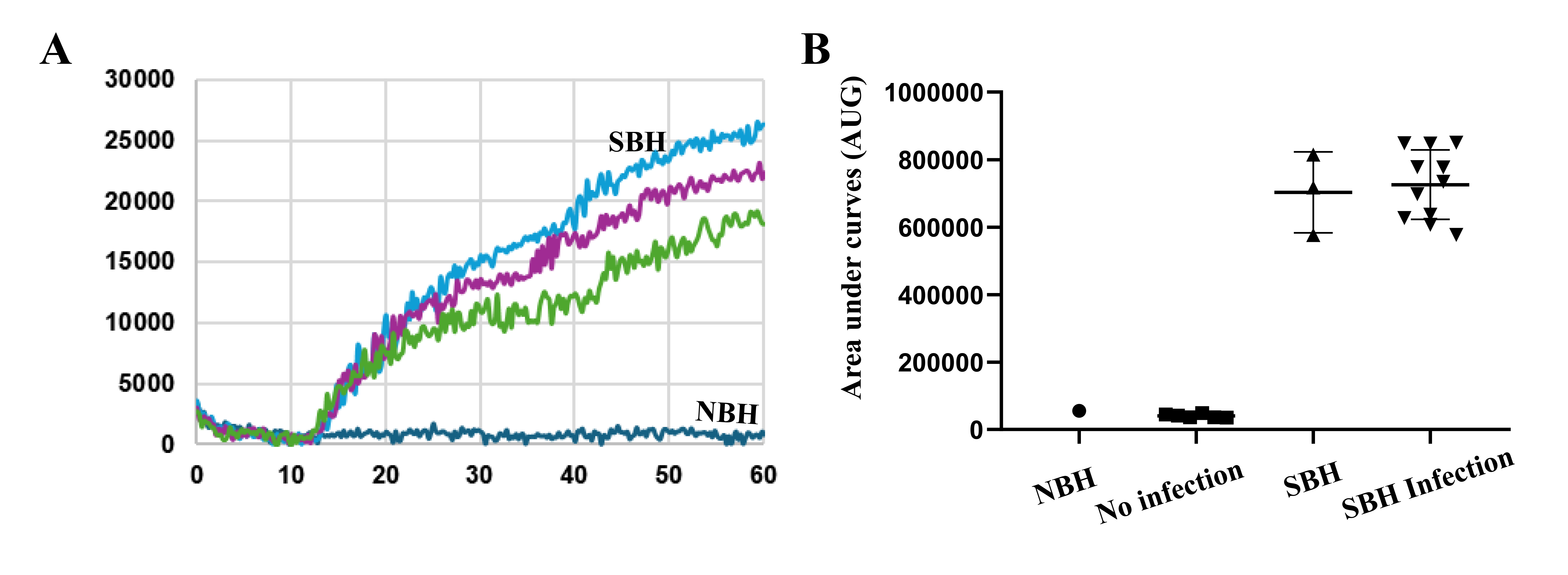


**Figure S3. RT-QuIC positive and negative controls and AUC-based comparison with infected spheroids.**
**(A)** RT-QuIC fluorescence curves obtained using normal brain homogenate (NBH) and sick brain homogenate (SBH) as negative and positive controls, respectively. SBH produced robust ThT fluorescence responses, whereas NBH remained at baseline levels.
**(B)** Quantification of RT-QuIC responses by area under the curve (AUC). The AUC of the SBH-infected spheroids was comparable to that of the positive control (SBH), whereas NBH and no-infection controls remained low.
